# Predicting foodborne pathogens and probiotics taxa within poultry-related microbiomes using a machine learning approach

**DOI:** 10.1186/s42523-023-00260-w

**Published:** 2023-11-15

**Authors:** Moses B. Ayoola, Nisha Pillai, Bindu Nanduri, Michael J. Rothrock Jr, Mahalingam Ramkumar

**Affiliations:** 1 Geosystems Research Institute, Mississippi State University, Starkville, MS 39762 USA; 2 Department of Comparative Biomedical Sciences, College of Veterinary Medicine, Mississippi State University, Starkville, MS 39762 USA; 3 Department of Computer Science and Engineering, Mississippi State University, Starkville, MS 39762 USA; 4 Egg Safety and Quality Research Unit, USDA-ARS U.S. National Poultry Research Center, Athens, GA 30605 USA

**Keywords:** Microbiome, Machine learning, Pastured poultry, Foodborne pathogens, Probiotics

## Abstract

**Background:**

Microbiomes that can serve as an indicator of gut, intestinal, and general health of humans and animals are largely influenced by food consumed and contaminant bioagents. Microbiome studies usually focus on estimating the alpha (within sample) and beta (similarity/dissimilarity among samples) diversities. This study took a combinatorial approach and applied machine learning to microbiome data to predict the presence of disease-causing pathogens and their association with known/potential probiotic taxa. Probiotics are beneficial living microorganisms capable of improving the host organism’s digestive system, immune function and ultimately overall health. Here, 16 S rRNA gene high-throughput Illumina sequencing of temporal pre-harvest (feces, soil) samples of 42 pastured poultry flocks (poultry in this entire work solely refers to chickens) from southeastern U.S. farms was used to generate the relative abundance of operational taxonomic units (OTUs) as machine learning input. Unique genera from the OTUs were used as predictors of the prevalence of foodborne pathogens (*Salmonella*, *Campylobacter* and *Listeria*) at different stages of poultry growth (START (2–4 weeks old), MID (5–7 weeks old), END (8–11 weeks old)), association with farm management practices and physicochemical properties.

**Result:**

While we did not see any significant associations between known probiotics and *Salmonella* or *Listeria*, we observed significant negative correlations between known probiotics (*Bacillus* and *Clostridium*) and *Campylobacter* at the mid-time point of sample collection. Our data indicates a negative correlation between potential probiotics and *Campylobacter* at both early and end-time points of sample collection. Furthermore, our model prediction shows that changes in farm operations such as how often the houses are moved on the pasture, age at which chickens are introduced to the pasture, diet composition and presence of other animals on the farm could favorably increase the abundance and activity of probiotics that could reduce *Campylobacter* prevalence.

**Conclusion:**

Integration of microbiome data with farm management practices using machine learning provided insights on how to reduce *Campylobacter* prevalence and transmission along the farm-to-fork continuum. Altering management practices to support proliferation of beneficial probiotics to reduce pathogen prevalence identified here could constitute a complementary method to the existing but ineffective interventions such as vaccination and bacteriophage cocktails usage. Study findings also corroborate the presence of bacterial genera such as *Caloramator, DA101, Parabacteroides* and *Faecalibacterium* as potential probiotics.

**Supplementary Information:**

The online version contains supplementary material available at 10.1186/s42523-023-00260-w.

## Background

Demand for poultry meat is growing exponentially as an affordable source of protein and it is currently the most consumed meat worldwide [1]. Chickens are the most efficient feed converter among the traditional livestock (such as pig, cattle and turkey) and this contributes to the increased demand for poultry products [2]. However, colonization of the chickens while on the farm and contamination of the final poultry product with zoonotic pathogens are of public health concern, oftentimes leading to shortage in supply and economic loss. Microbial colonization of newly hatched chicks is reported to start at the hatching stage, but there is a possibility that it could be earlier as evidenced by the passage of pathogens through the egg pores [3]. Strict hygiene measures are usually implemented in commercial poultry to reduce colonization of eggs and newly hatched chickens by microbes from the environment, but further evidence suggests that inheritance of the colonizers from the parent is possible at the embryonic stage [4].

The chicken gut helps maintain intestinal homeostasis by competitively excluding pathogens or preventing colonization. This competitive exclusion is expected to lower the energy required to maintain the immune system and ultimately improve chicken performance [5]. Prophylactic use of antibiotics as part of management practice or abuse of antibiotics to treat infections can lead to imbalance in the microbiome, drug resistance and exacerbation of infection. Due to the growing concern regarding the transfer of antibiotic resistance from animals to human, the United States banned the use of antibiotics as growth promoters in livestock farming, including poultry [6], which mandates the development of alternate strategies to limit the prevalence of pathogens in the production environment.

Dysbiosis in the chicken gut microbiome could affect intestinal microbiota, which in turn could impact immunity, digestion and intestinal integrity, which in turn affect energy available to the chicken host and have been established as a function of microbiome composition and diversity [7, 8]. Currently, beneficial microbes such as probiotics are being directly applied to the chicken or inside the egg for creating a healthy microbiome as a suitable alternative to antibiotics in both poultry and alternative poultry systems for animal welfare [9, 10]. Increased poultry growth, performance and immune response, as well as improved meat and egg quality, have been associated with healthy microbiomes nurtured by probiotics, but their usage is still largely at the evaluation stage as a suitable alternative to antibiotics usage [11]. Identifying relationships between management practices, impact on gut microbiome (including probiotics) and pathogen prevalence in pastured poultry management systems, where antibiotics are not used historically, could provide useful information on appropriate probiotic intervention mechanisms.

The application of machine learning to find biologically relevant patterns from large datasets is becoming common in many domains, including microbiome data analysis. The random forest algorithm has been successfully used to find relationships between human gut microbiomes and disease states such as atherosclerosis, diabetes and arthritis [12]. Associations between gut microbial single nucleotide variants and colorectal cancer have also been predicted and established using the random forest algorithm [13]. The application of machine learning tools enables analyses to move beyond the routine alpha- and beta-diversity comparisons often associated with microbiome data. This study generated microbiome datasets from pre-harvest (feces, soil) poultry-related samples from 42 pastured poultry flocks in the southeastern U.S. and (1) focused on how changes in the microbiome of temporal pastured poultry pre-harvest samples could help predict prevalence of common poultry pathogen species such as *Salmonella*, *Campylobacter* and *Listeria* in the poultry environment, (2) examined their associations with known and potential probiotics in the microbiome and (3) determined how these changes are related to different farm management practices and physicochemical properties of the farms. We initially employed three different machine algorithms, random forest (RF), support vector machine (SVM) and logistic regression (LogReg), for training and evaluation purposes to access their suitability for our microbiome and pastured poultry data. Cross-validation analysis of the results indicated that RF performed optimally in learning from training data and making the best prediction about the evaluation data, and this algorithm was employed for the downstream analysis of predicting presence of pathogens, associated changes in farm management practices and sample physicochemical levels.

## Results

### Microbiome and poultry data

A total of 1,393 feces and soil samples were collected at different times throughout the chicken growth cycle (Feces_START (2–4 weeks old) = 200, Feces_MID (5–7 weeks old) = 313, Feces_END (8–11 weeks old) = 185, Soil_START (2–4 weeks old) = 199, Soil_MID (5–7 weeks old) = 313, Soil_END (8–11 weeks old) = 183) from eleven pastured farms in the southeastern United States. DNA was extracted from all the samples and the V4 domain of the bacterial 16 S rRNA gene was amplified. After sequencing and preliminary analysis using the QIIME pipeline, 1,823 microbiome operational taxonomic units (OTUs) were identified. During the data preprocessing stage, the dimension of the OTUs were reduced to 877 unique genera. These microbiome genera were used as input to predict the prevalence of 3 important foodborne pathogens (*Salmonella*, *Campylobacter* and *Listeria*) that were culturally isolated, levels of physicochemical properties (acidity/basicity (pH), electrical conductivity (EC), moisture, total carbon (TotalC), total nitrogen (TotalN), carbon to nitrogen ratio (CNRatio), aluminum (Al), boron (B), calcium (Ca), cadmium (Cd), chromium (Cr), copper (Cu), iron (Fe), potassium (K), magnesium (Mg), manganese (Mn), molybdenum (Mo), sodium (Na), nickel (Ni), phosphorus (P), lead (Pb), sulphur (S), silicon (Si), zinc (Zn)) and changes in farm management practices associated with the samples as machine learning targets. The 32 farm management practices (AvgNumBirds, AvgNumFlocks, YearsFarming, EggSource, BroodBedding, BroodFeed, BrGMOFree, BrSoyFree, BrMedicated, BroodCleanFrequency, AvgAgeToPasture, PastureHousing, FreqHousingMove, AlwaysNewPasture, PastureFeed, PaGMOFree, PaSoyFree, PaMedicated, LayersOnFarm, CattleOnFarm, SwineOnFarm, GoatsOnFarm, SheepOnFarm, WaterSource, FreqBirdHandling, AnyABXUse, LengthFeedRestrixProcess, Seasons, FlockAgeDays, Breed, FlockSize, AnimalSource) have been previously described [14, 15].

### Model evaluation

Cross-validation is a generally accepted resampling technique suitable for machine learning model evaluation as it trains on different parts of the data and evaluates prediction accuracy on the rest of the data in many iterations. Out of the three algorithms tested with microbiome relative abundance as the input and different pathogens, farm practices and physicochemical properties as targets, random forest performed optimally the best with the average 5-fold cross-validation of 0.83, followed by logistic regression with 0.76 and support vector machine with 0.65 average performance (data not shown). With 59 target variables, 47 models performed relatively well with RF, above 70% confidence threshold with 5-fold cross-validation (Table 1) while 12 models performed poorly with less than 70% with 5-fold cross-validation (Supplementary Table 1). Definitions of these variables have been previously published [14, 15].

**Table 1:** Comparison of random forest (RF), support vector machine (SVM) and logistic regression (LogReg). Model performance was considered good when 5-fold cross-validation value ≥ 0.7

Number	Target Variables	RF	SVM	LogReg
	Pathogens			
1	*Salmonella*	0.85	0.85	0.78
2	*Campylobacter*	0.80	0.65	0.74
3	*Listeria*	0.86	0.86	0.79
	**Common Farm Practice Variables**			
4	BroodBedding	0.91	0.91	0.88
5	BrGMOFree	0.78	0.69	0.72
6	BrSoyFree	0.84	0.83	0.8
7	BrMedicated	0.97	0.97	0.94
8	AvgAgeToPasture	0.73	0.61	0.71
9	PastureHousing	0.72	0.55	0.62
10	FreqHousingMove	0.98	0.98	0.96
11	AlwaysNewPasture	0.9	0.87	0.86
12	PaGMOFree	0.77	0.67	0.73
13	PaSoyFree	0.76	0.64	0.71
14	PaMedicated	0.98	0.98	0.95
15	LayersOnFarm	0.95	0.95	0.93
16	CattleOnFarm	0.77	0.6	0.7
17	SwineOnFarm	0.83	0.8	0.81
18	GoatsOnFarm	0.74	0.63	0.7
19	SheepOnFarm	0.78	0.55	0.7
20	WaterSource	0.7	0.52	0.62
21	FreqBirdHandling	0.9	0.87	0.86
22	AnyABXUse	0.98	0.98	0.95
23	AnimalSource	0.90	0.85	0.87
	**Physicochemical Properties**			
24	pH	0.93	0.56	0.81
25	EC	0.96	0.65	0.89
26	Moisture	0.97	0.66	0.91
27	TotalC	0.94	0.6	0.85
28	TotalN	0.94	0.63	0.83
29	CNRatio	0.98	0.55	0.82
30	Al	0.92	0.55	0.73
31	B	0.94	0.58	0.77
32	Ca	0.95	0.63	0.84
33	Cd	0.98	0.65	0.92
34	Cr	0.98	0.9	0.92
35	Cu	0.94	0.6	0.84
36	Fe	0.95	0.61	0.87
37	K	0.97	0.55	0.9
38	Mg	0.95	0.69	0.85
39	Mn	0.91	0.63	0.81
40	Mo	0.98	0.7	0.92
41	Na	0.96	0.68	0.88
42	Ni	0.96	0.65	0.89
43	P	0.97	0.67	0.89
44	Pb	0.91	0.55	0.79
45	S	0.94	0.57	0.78
46	Si	0.93	0.54	0.73
47	Zn	0.92	0.64	0.85

### Feature importance

**Fig. 1:**
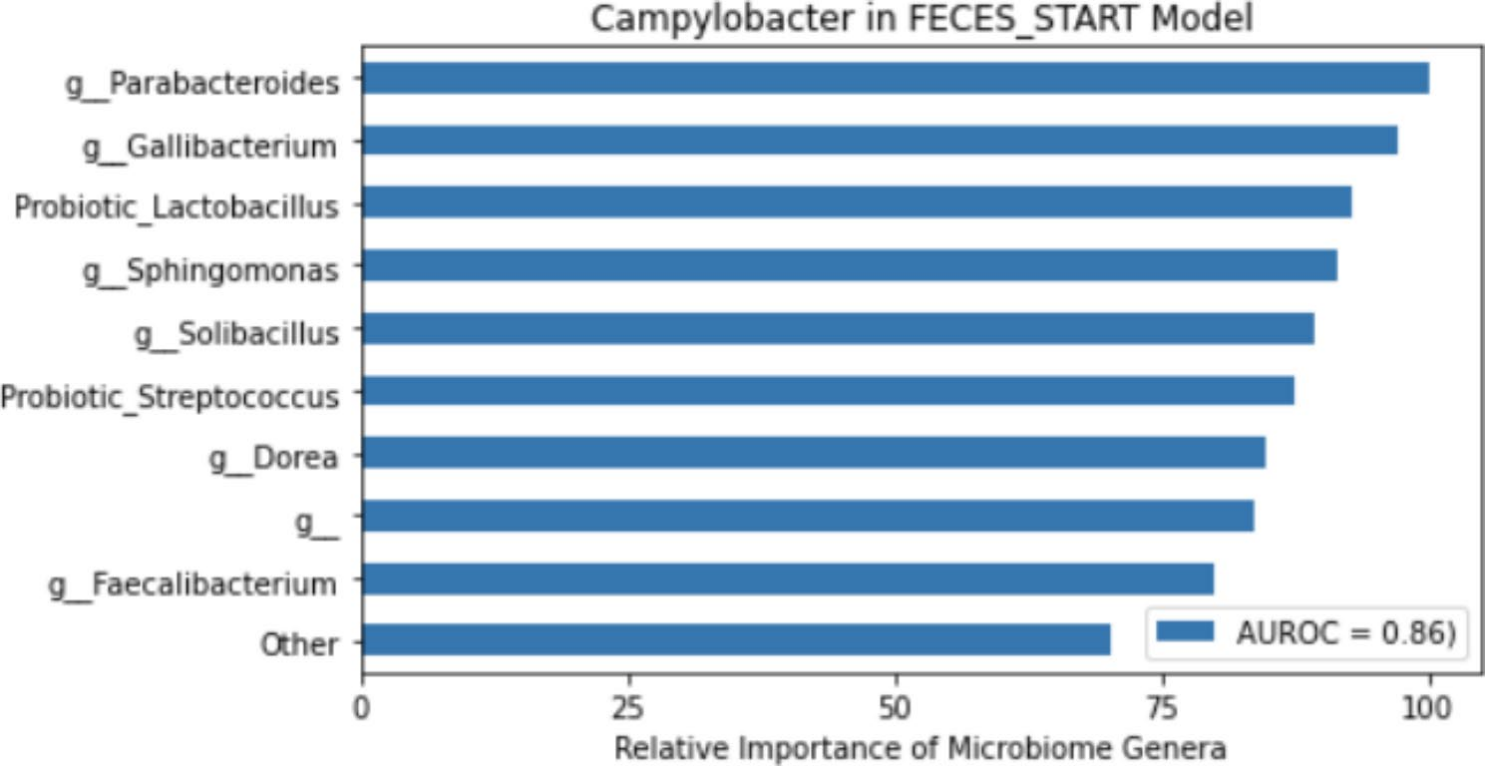
Representative feature importance plots showing the top 10 genera (including probiotic genera) that are important for the prediction of *Campylobacter* in the feces samples when the flock is first introduced onto the pasture (Feces_START). The plot ranks the genus with the highest importance score as 100

Using the random forest feature importance package in scikit-learn library (v0.24.2), which is based on impurity decrease within each decision tree, we identified the top 10 important microbiome genera associated with each target variable. A representative plot showing the top 10 important genera out of a total of 877 genera that are associated with *Campylobacter* positive samples (as determined using traditional cultural protocols) is shown in Fig. 1. The corresponding area under the receiver operating characteristic curve (AUROC) is indicated on the plot and any prediction model with AUROC < 0.70 was discarded.

### Correlation of pathogens and probiotics

After successfully classifying the top 10 genera that predict the presence of *Salmonella* spp., *Campylobacter* spp. and *Listeria* spp. in different samples, we focused on genera among the top 10 that are identified in literature as probiotics such as *Bacillus*, *Bifidobacterium*, *Clostridium*, *Enterococcus*, *Lactobacillus*, *Pediococcus*, *Propionibacterium* and *Streptococcus* for further analysis. We used the Pearson statistical module in SciPy package (v1.7.0) to identify a significant relationship (*r*) between the identified probiotics and pathogens (*p* < 0.05). This correlation analysis is expected to yield useful information on potentially suitable probiotic taxa within pastured poultry management systems. No significant negative associations were found between *Salmonella-* and *Listeria-*positive samples and any of the known probiotics and no relationships were found between probiotics and the pathogens of interest in the soil sample type. Therefore, all subsequent analyses focused on *Campylobacter*-positive feces samples. *Campylobacter*-positive feces samples were negatively correlated with both probiotics *Bacillus* (*r* = -0.77, *p*-value = 3 × 10^− 21^) and *Clostridium* (*r* = -0.82, *p*-value = 7.0 × 10^− 26^) and positively correlated with *Lactobacillus* (*r* = 0.83, *p*-value = 5.0 × 10^− 27^).

**Fig. 2:**
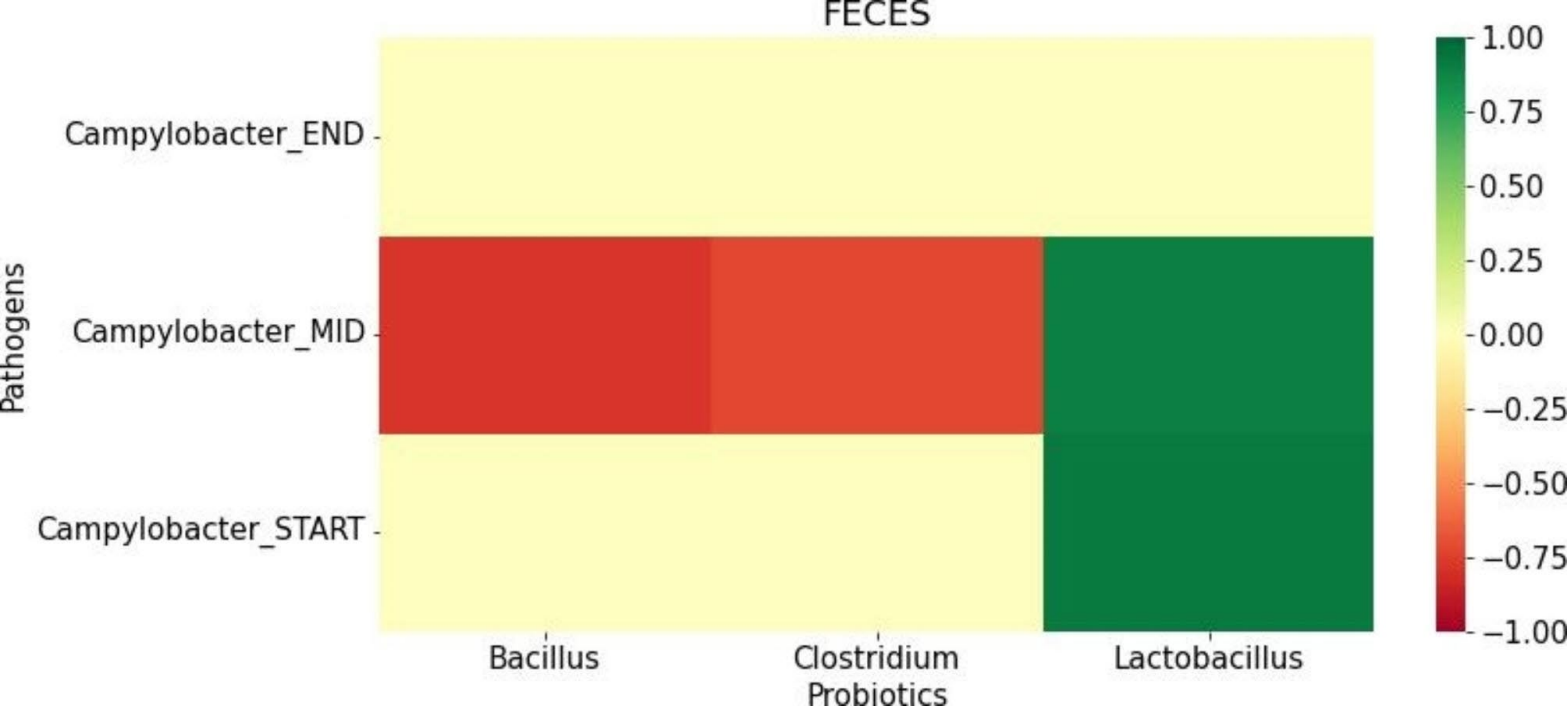
Heatmap showing the correlation of probiotic taxa with *Campylobacter*-positive feces at different stages of pastured poultry production (START, MID, END). Strong positive correlations (*r* ≥ 0.7, *p*-value < 0.05) are depicted in different shades of green while strong negative correlations (*r* ≤ -0.7, *p*-value < 0.05) are shown in different shades of red. A strong negative correlation is observed with both *Bacillus* and *Clostridium* taxa in *Campylobacter*-positive feces at the second time point (MID) of feces collection. Probiotic taxa *Lactobacillus* is positively correlated to *Campylobacter-*positive feces at both START and MID time points of sample collection

Following the identification of relationships between *Campylobacter*-positive samples and probiotic taxa *Bacillus*, *Clostridium* and *Lactobacillus* in the fecal microbiomes of those samples, we used RF to build new models based on the time of sample collection to identify the time point at which these correlations actually occur as this information will help identify at what flock age potential probiotic application would be most impactful. Using seaborn package (v 0.10.1), our observation is presented as the heatmap in Fig. 2.

### Correlation of *Campylobacter* and other genera

**Fig. 3:**
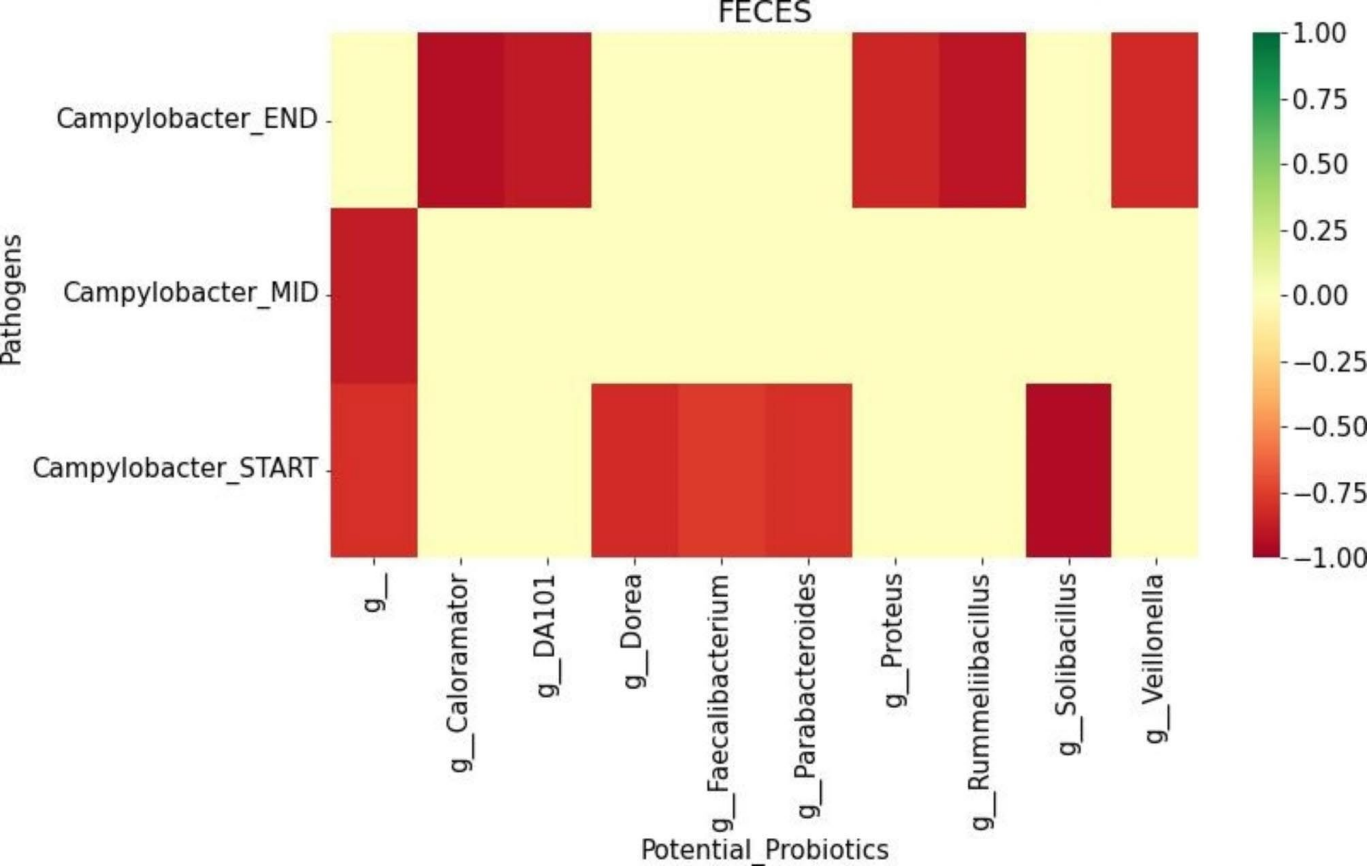
Heatmap showing the correlation between “non-probiotics” microbiome taxa and *Campylobacter*-positive feces samples. All genera observed in the analysis have a negative correlation with *Campylobacter*-positive feces samples at the start and end-time point of sample collection. The only negative correlation identified at the mid-time point belongs to the group of OTUs that could not be identified at the genus level (g_). Strong negative correlations (*r* ≤ -0.7, *p*-value < 0.05) are depicted in red

The correlations between *Campylobacter-*positive fecal samples and other “non-probiotics” taxa identified among the top 10 in feces at different time points of collection using RF, its feature importance package and Pearson correlation analysis (Fig. 3) are discussed in this section. While taxa that are negatively correlated with *Campylobacter* are potentially novel probiotics, a positive correlation of a particular genus could serve as an indicator species that forewarns the presence of *Campylobacter*. We identified genera such as *Dorea*, *Faecalibacterium*, *Parabacteroides* and *Solibacillus* that are negatively correlated with *Campylobacter* at the START phase and *Caloramator*, *DA101*, *Proteus*, *Rumellibacillus* and *Veillonella* that are also negatively correlated at the END production phase. The only correlation identified at the MID phase belongs to taxa with uncertain genera. We did not identify any positive correlation between the “non-probiotics” taxa and *Campylobacter* that could serve as indicators.

### Correlation of farm management practices, feces physicochemical properties and probiotics

**Fig. 4:**
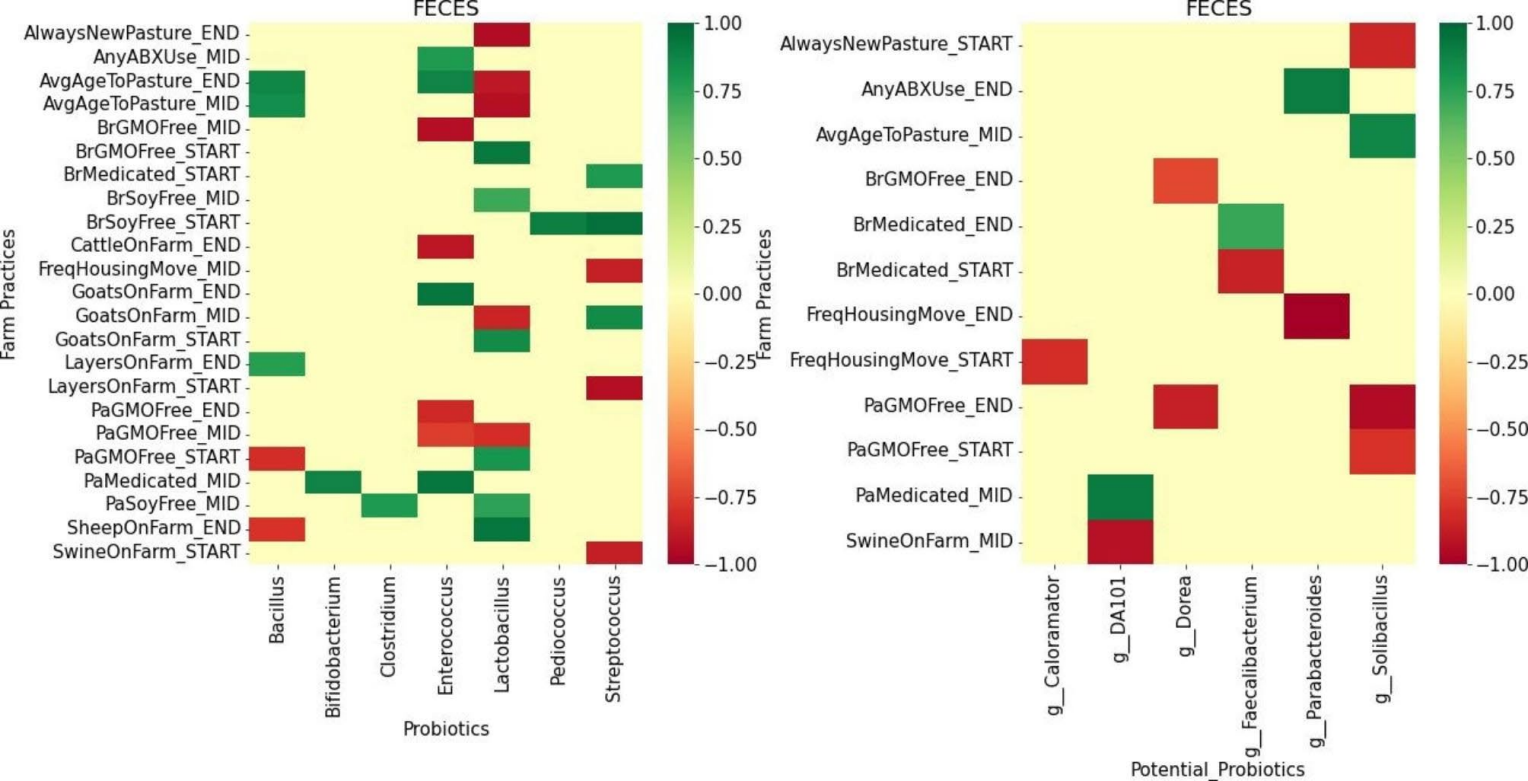
Heatmap showing the correlation of common/potential probiotic taxa associations with diverse farm management practices. Strong positive correlations (*r* ≥ 0.7, *p*-value < 0.05) are shown in different shades of green while strong negative correlations (*r* ≤ -0.7, *p*-value < 0.05) are shown in different shades of red. There is a negative correlation between most known probiotics and farm practices such as AlwaysNewPasture, FreqHousingMove, PaGMOFree, CattleOnFarm and SwineOnFarm and a positive correlation with BrSoyFree, PaSoyFree and GoatOnFarm. Similar to our observation with known probiotics, FreqHousingMove, PaGMOFree and SwineOnFarm have a negative correlation with potential probiotics

**Fig. 5:**
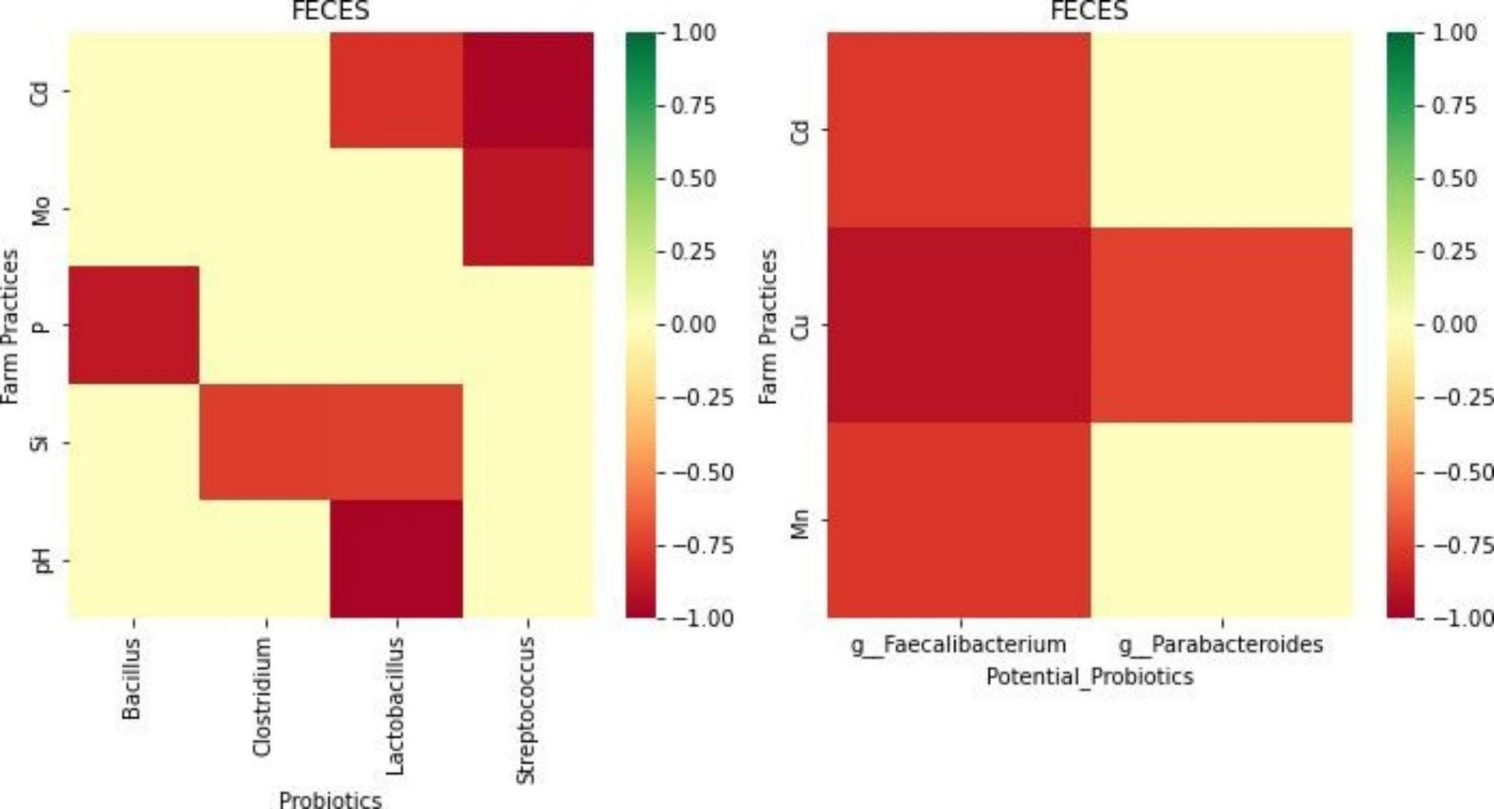
Heatmap showing the correlation between common/potential probiotic taxa associations with physicochemical properties in feces. All association between physicochemical elements of feces are negatively correlated (*r* ≤ -0.7, *p*-value < 0.05) to the common probiotics and potential probiotics

Once we established that there is a correlation between the microbiome (probiotics and other non-probiotics genera) with *Campylobacter*, we next identified farm management practices and feces physicochemical properties that are associated with these microbiome signatures that correlate with *Campylobacter* by building RF models. We used the relative abundance for all taxa at the genus level of the microbiome genera from 698 feces samples as input to RF models with different farm management practices and feces physicochemical properties as the individual targets. Using random forest feature importance, Pearson correlation, and scikit-learn seaborn package, we identified associations between known probiotics and potential probiotic taxa (identified in the previous section) and farm management or physicochemical properties (Figs. 4 and 5, respectively). Predicting associations between probiotic taxa and *Campylobacter* prevalence, based on cultural identification (Figs. 2 and 3) as well as probiotic taxa and farm management or fecal physicochemical properties (Figs. 4 and 5, respectively) from the same input microbiome could identify management practices or physicochemical properties that could be modified to optimize a healthy microbiome and selectively target *Campylobacter* and reduce its prevalence. We observed that GMO free feed and presence of sheep on the farm have a negative correlation with the level of *Bacillus* while GMO free feed and having cattle on the farm have a negative correlation with *Enterococcus* (Fig. 4). Keeping brood and pasture feeds free of soy products, especially at the START and MID phases of production could potentially increase the abundance of *Clostridium*, *Lactobacillus*, *Pediococcus* and *Streptococcus*. Always moving the chicken to new pasture without rotating back to previously used farm lots, putting chicks on pasture later at 4 weeks instead of 3 weeks, having goats on the farm, and feeds that are GMO free could all negatively impact the abundance of *Lactobacillus.* The presence of swine and layers on the farm and frequently moving pasture housing everyday instead of every 2 days may negatively impact the abundance of *Streptococcus*. Some farm management practices such as AvgAgeToPasture and SheepOnFarm have both negative and positive correlations, depending on the probiotic genus. For example, AvgAgeToPasture has a negative correlation with *Lactobacillus* but a positive correlation with *Bacillus*. GMO free diets, moving the pasture house daily and having other animals such as swine on the farms appears to not only negatively impact common probiotics but the potential probiotics (*DA101, Dorea, Parabacteroides* and *Solibacillus*) as well (Fig. 4).

Looking at the relationship between physicochemical properties and probiotics, four elements identified (Cd, Mo, P, S) and pH have a negative correlation with known probiotics, mostly *Lactobacillus*, while three elements (Cd, Cu, Mn) have a negative correlation with potential probiotics, especially *g_Faecalibacterium*, which has a negative association with all the three elemental metals (Fig. 5). All association between physicochemical elements of feces are negatively correlated (*r* ≤ -0.7, *p*-value < 0.05) to both the common probiotics and potential probiotics. Cadmium (Cd) in particular negatively impacts *Lactobacillus*, *Streptococcus*, and *g_Faecalibacterium*, while copper (Cu) also has a negative correlation with *g_Faecalibacterium* as well as *g_Parabacteroides*.

## Discussion

With the growing capability of artificial intelligence to find patterns in large datasets, machine learning has found applications in the biomedical research in diverse areas such as cancer detection, drug development, treatment recommendation and prediction of disease outcome ﻿[16, 17]. Machine learning is currently being explored for a quick turnaround in finding association between disease states, causative agents and the grey areas in between. Traditional biological research to elucidate such associations usually takes years, requires exorbitant funding and is oftentimes laborious. Interactions between bacterial pathogen (*Yersinia pestis, Bacillus anthracis* and *Francisella tularensis*) proteins and human proteins have been successfully predicted by machine learning [18]. Relationships between microbiome dysbiosis and diseases such as ulcerative colitis, obesity and amyotrophic lateral sclerosis in humans has been established [19–21]. Traditional machine learning algorithms, such as support vector machine, random forest and logistic regression, have been used to identify available microbiome features like relative abundance that is linked to obesity and diabetes [22]. Random forest, a popular machine learning algorithm, has been used to identify structural features of beta-glucans, making it suitable for developing prebiotics [23].

Probiotics which are microorganisms from a healthy host are being considered in the food safety industry as they are carefully chosen for their non-pathogenic nature and ability to confer health benefits to the new host they are administered to. Although the exact mechanism of action is not well understood and often is strain dependent, improving host nutrient absorption, secretion of toxins such as bacteriocin, altering pH of the intestinal lumen, production of short-chain fatty acids, modulation of the immune response and competing for nutrients are some of the ways in which probiotics have been proposed to competitively exclude the pathogens and benefit both humans and animals [24, 25]. While the weight gain benefit of probiotics in livestock has been reported [26], their ability to reduce contamination of pathogens such as *Salmonella* spp. in chickens, *E. coli* in pigs and *Aeromonas salmonicida* in fish and improved food safety has also been recorded [27]. The benefits of probiotics in conventional poultry have been documented and a similar positive effect has been proposed in the alternative poultry production systems [10]. However, baseline level and dynamics of these useful microorganisms which are part of the natural microflora have not been established in pastured poultry.

In this study, microbiome relative abundance values were used as input to the RF algorithm to predict the presence of enteric poultry foodborne pathogen species (*Salmonella*, *Campylobacter* and *Listeria*), find possible relationships with known probiotic genera (*Bacillus, Bifidobacterium, Clostridium, Enterococcus, Lactobacillus, Pediococcus, Propionibacterium, Streptococcus* [28, 29]) and identify novel genera that represent potential probiotics [30] in a time dependent manner based on different stages of microbiome sample collection across poultry production phases. Samples from which the relative abundance was generated include feces as a representation of the gut microbiome in pastured poultry and soil as a representation of communities of microbes in the poultry environment. Existing literature on microbiome analysis usually focus on alpha diversity, such as measures of microbiome richness and evenness within samples, and beta diversity, dissimilarities among microbiome samples to identify prevalent OTUs or attribute them with certain disease conditions [31, 32]. This approach is inadequate to answer the question about the correlation between enteric pathogens and those found in the poultry environment with respect to the level of the probiotics within the microbiome as an innate defense system. Here, using the machine learning approach, we wanted to establish if microbiome data could be used successfully to predict pathogen prevalence, and if so, is there a correlation between the pathogens and the probiotics contained in the microbiome. A similar approach using random forest and relative abundance feature has been used to find association between the microbiome and disease conditions such as cirrhosis, colorectal cancer, diabetes and obesity [22]. Furthermore, we explored the relationship between the probiotics and different farm management practices and physicochemical properties. We hypothesized that certain practices and/or properties could be altered to enhance the abundance of probiotics and potentially reduce pathogen prevalence.

Of the three poultry foodborne pathogens (*Salmonella*, *Campylobacter* and *Listeria*) and two sample types (feces and soil) specifically targeted in this study, the machine learning models were only able to identify significant associations for *Campylobacter*-positive feces samples. *Campylobacter*, the zoonotic causative agent of campylobacteriosis and gastroenteritis, was the predominant foodborne pathogen identified in all the eleven pastured poultry farms sampled in this study with negative correlations to both known and potential probiotics (Figs. 2 and 3). *Campylobacter* is a thermophilic bacterium that colonizes chicken due to their higher body temperature. It is often found in farmhouses, poultry house water and even capable of surviving on slaughtering equipment despite sanitization. *Campylobacter* has a high infection rate, with about 95% of birds becoming infected within 4 to 7 days after colonization of the first broiler [33]. Due to controlled use of antibiotics to curb the growing trend of antibiotic resistance and failure of several physical biosecurity and hygiene measures to prevent *Campylobacter* contamination of poultry product [34], vaccination, bacteriocins, bacteriophage cocktails and probiotics administrations are being proposed as alternatives to control this pathogen [35]. Supplementation with probiotics, which are a natural part of the chickens’ microbiome, is being suggested as the most viable alternative as they could not only competitively exclude the pathogen but also enhance immune response and improve digestibility and nutrient absorption [36, 37]. We observed that the prevalence of *Campylobacter* detection by the cultural isolation method is higher in both feces and soil samples when the poultry chickens were young and old than at mid-age of their lifetime (Supplementary Fig. 1), suggesting that age may play a role in colonization by *Campylobacter* spp. Colonization of chicken with *Campylobacter* has been reported to start in the first three to four weeks of life [38]. And similar to our observation, Babacan et al. showed higher *Campylobacter* colonization towards the old-age (around 7 to 8 weeks) than at mid-age (5 to 6 weeks) [39]. The interpretation of this finding is that probiotics containing these taxa could be administered when the chicks are newly introduced to the pasture when their gut microbiomes are still maturing and re-applied closer to slaughter time to potentially control *Campylobacter* before entering the processing/post-harvest stage. Identifying the best time of probiotic application will also invariably reduce production costs in terms of the amount of probiotics required to be administered. Interestingly, our analysis identifies a strong negative correlation between probiotics *Bacillus* and *Clostridium* and *Campylobacter* at mid-age of the chickens (Fig. 2). Based on these findings, we recommend administration of existing probiotics, especially *Bacillus* and *Clostridium*, preferably at the early age of the chicks and late age as a suitable strategy to reduce *Campylobacter* contamination in poultry.

*Lactobacillus*, *Bacillus* and *Bifidobacterium* species as probiotics have been previously used to inhibit the growth and reduce the virulence of *Campylobacter* [40]. Our analysis predicts negative associations between *Campylobacter* and common probiotics *Clostridium* and *Bacillus* as expected, however it identified a positive association with *Lactobacillus*. Although most *Lactobacillus* species act as probiotics, certain species such as *L. rhamnosus* have been positively associated with several infections [41]. Since the microbiome analysis in this study could only identify the taxa down to the genus level, this could possibly explain the positive association between *Campylobacter* and an unknown strain of *Lactobacillus* that could be pathogenic such as *L. rhamnosus*, an indicator species for *Campylobacter*. In addition, efforts to identify other microorganisms, other than the conventional ones, that could be beneficial as probiotics are ongoing in agriculture and food industry [42]. In aquaculture, *Lysinibacillus macrolides* was recently shown to be a potential probiotic as it significantly helped to improve the growth rate and weight gain of *Cyprinus carpio* fish [30]. In this study, we identified 9 different genera such as *Caloramator*, *DA101*, *Dorea*, *Faecalibacterium*, *Parabacteroides*, *Proteus*, *Rumellibacillus*, *Solibacillus* and *Veillonella* in a feces model (Fig. 3) that could potentially be considered as probiotics against *Campylobacter*. Existing literature shows that *Caloramator* is closely related to probiotic *Clostridium* as they both belong to the same family, *Clostridiaceae* [43]. Also, positive roles of *Caloramator* in regulating inflammation and the immune system and promotion of gut health have been described in broiler chickens [44]. Supplementation with probiotic *Bacillus* increased the proportion of *DA101* and significantly improved the performance of the broiler chickens [45], suggesting that genus *DA101* could also potentially be a probiotic. *Faecalibacterium* was recently shown as both a biomarker of obesity and a probiotic in treatment of this condition [46] while *Parabacteroides* has been proposed as a next-generation probiotic [47]. The potential of *Solibacillus* as a probiotic against pathogens such as *Aeromonas* and *Pseudomonas* has been adequately demonstrated [48]. Additionally, the ability of *Veillonella* to effectively inhibit the growth of *Salmonella enteritidis* by producing acetate and propionate intermediates has also been reported [49]. In summary our model has identified not only known probiotics to target as potential interventional strategies against *Campylobacter*, but also discovered other taxa with known probiotic potential for consideration in future studies to validate their beneficial role in commercial poultry.

In evaluation of different farm practices that could impact the abundance of probiotic taxa in the pastured poultry gut microbiome (as represented by feces samples), farm management variables had a greater impact on the known probiotic taxa than the identified potential probiotics (Fig. 4). It should be stated that the variability of management practices on pastured poultry farms is much greater than what is observed on conventional poultry farms, with the broiler flocks having much greater interaction with the farm environment and the other animals being raised on those farms. Out of the eight probiotics commonly used in poultry, only *Bacillus*, *Enterococcus*, *Lactobacillus* and *Streptococcus* are mostly affected by the farm activities in this study. We observed that the presence of sheep and a GMO-free diet have a negative correlation with the relative abundance of *Bacillus* within the broiler fecal microbiome, while having layers on the farm was associated with higher *Bacillus* abundance (Fig. 4). Our model suggests that soy-free diets have significantly positive correlation with the abundance of *Clostridium, Lactobacillus, Pediococcus* and *Streptococcus* in the fecal microbiome. While a soy-free diet may improve the abundance of probiotics, it has also been shown to reduce the microbiome relative abundance of pathogens such as *Campylobacter* and *Acinetobacter* [50]. GMO-free feed and the presence of cattle on the farm negatively impact *Enterococcus* abundance while having goats on the farm and delaying the age chickens are put on pasture from 3 weeks to 4 weeks could increase its abundance. Similar to previous observation, goats on the farm appear to improve the abundance of S*treptococcus* while layers and swine on the farm reduce the abundance of *Streptococcus* within the poultry fecal microbiome. While the mechanisms are not clearly understood at this time, the presence of goats with the poultry, a soy-free diet and increasing the age to put chickens on pasture from 3 weeks to 4 weeks should help improve the abundance of these probiotics. Having swine and cattle on the farm, a GMO-free diet and daily moving of pasture houses may be discouraged as they do not just negatively impact the abundance of the known probiotics alone but the potential novel probiotics as well.

Out of the 24 physicochemical properties modeled as machine learning targets, we observed that the presence of six elements (Cd, Cu, Mn, Mo, P, and Si), especially Cd, Cu and Si, have a varying degree of negative impacts on the relative abundance of probiotic taxa in the fecal microbiome data (Fig. 5). Literature pertaining to the exact mechanism of action of these elements in modulating the probiotics activities is very limited. The ability of *Lactobacillus* and *Streptococcus* as probiotics to effectively bind Cd as a heavy metal and source of oxidative stress and reduce its toxicity has been demonstrated in both mice and rats [51–53]. Cd has been shown to significantly reduce *Lactobacillus* and microbiome abundance in general [54]. Similar to our finding here, Zhang and colleagues show that an increased level of Cu decreases the abundance of genus *Parabacteroides* in the rat microbiome [55] while the ability of *Faecalibacterium* in the microbiome to detoxify arsenic, often found in complexes with Cu, has been demonstrated [56]. In contrast, Si, with its antioxidant properties, is reported to have positive correlation with beneficial *Lactobacillus reuteri* and *Lactobacillus murinus* [57]. Si has a negative correlation with *Clostridium* and *Lactobacillus* in our dataset, although specific *Lactobacillus* species that have negative correlation could not be ascertained.

## Conclusion

In this study we describe a combinatorial approach using machine learning and microbiome relative abundance for predicting pathogens prevalence, identifying probiotics (*Bacillus* and *Clostridium*) suitable for control of *Campylobacter*, validating potential probiotics candidates (*Caloramator*, *DA101*, *Dorea*, *Faecalibacterium*, *Parabacteroides*, *Proteus*, *Rumellibacillus*, *Solibacillus* and *Veillonella*) and identifying farm management practices and farm physiochemical properties that may affect the probiotic strains that could limit the spread of *Campylobacter* along the pastured poultry farm-to-fork continuum. Although we initially considered checking for associations between microbiome taxa and different individual farms, the total number of samples collected is not evenly distributed among individual farms, making it difficult to apply machine learning algorithms such as RF to effectively train and learn from individual farms. Therefore, in this study, we combined all samples into a single comprehensive dataset. However, we acknowledge that Farm “A” and “I,“ which account for 18 out of 42 flocks (i.e., 43% of the total flocks) studied (Table 2), could have correlated observations that were not considered by RF due to the assumption of independence. Despite this limitation, it is important to note that while the total number of samples in this animal study (1393 samples) may be fewer than those reported in similar human studies (4347 samples [12] and 2424 samples [22]), our results offer valuable insights and generate hypothesis regarding microbiome signatures that could reduce pathogen prevalence and the impact of farm practices on these potential beneficial microorganisms for food safety. These results demonstrate the utility of this combinatorial approach with microbiome datasets for predicting microbiome signatures that could be targeted for developing “all natural” probiotic-based interventions for reduction of pathogens within pastured poultry management systems.

## Materials and methods

### Flock management, sample collection and preparation

This study was conducted in eleven pasture-raised broiler farms located in the southeastern United States. Flock sizes range between 25 and over 1500 with Freedom Ranger and Cornish Cross breeds being placed in different farms (Table 2). Soil and feces samples were collected from the pasture where the flock was currently residing at the time of sampling. Samplings occurred within a few days of being placed in the pasture (START = 2–4 weeks old), halfway through their time on pasture (MID = 5–7 weeks old), and on the day the flock was processed (END = 8–11 weeks). At each sampling time, the pasture area was divided into five separate sections and five subsamples in each section were pooled into a single sample for each section (a total of five soil samples and five feces samples were collected on each sampling day). Soil samples were collected from the surface (0–7 cm) with sterile scoops, and feces samples were collected from fresh droppings on the soil surface. Gloves and scoops were changed between sample types and between sampling areas. To prepare the environmental samples for homogenization, 3 g (feces, soil) were combined within filtered stomacher bags (Seward Laboratory Systems, Davie, FL, USA), and diluted 1:3 using 10 mM phosphate-buffered saline (PBS). All fecal and soil samples were transported back to the laboratory on ice and processed within 2 h of collection. All samples were homogenized for 60 s and these homogenates were used for all downstream cultural isolations and DNA extraction.

**Table 2:** Comparison of the 11 antibiotic-free pastured broiler farms in this study

Farm	Breed^1^	No. of flocks	Flock size	Multi-use farm?	Animal type
A	FR	10	> 500	Yes	Layers, swine, cattle, sheep
B	FR, CC	5	< 50	Yes	Layers, swine, goats
C	FR	1	< 50	No	NA
D	FR	1	< 50	No	NA
E	FR, CC	5	50–100	Yes	Layers, swine, cattle, sheep
H	FR	2	> 500	Yes	Layers
I	FR, CC	8	100–500	Yes	Layers, swine, goats
J	FR, CC	2	50	Yes	Layers
K	FR	4	100–500	Yes	Layers, cattle, goats
L	FR	2	> 500	Yes	Layers, swine, cattle, sheep
M	CC	2	50–100	Yes	Layers, swine

^1^ FR = Freedom Ranger, CC = Cornish Cross

### Cultural isolation methods

To determine the foodborne pathogen status for each fecal and soil sample in the study, the following traditional cultural isolation protocols were used.

#### Salmonella spp

As a pre-enrichment step, the stomached homogenates remained in the filtered stomacher bags and were incubated overnight at 35 °C. Two different enrichment broths were used to isolate *Salmonella spp.* from these environmental samples: Tetrathionate (TT; Becton Dickinson, Sparks, MD, USA) broth and Rappaport-Vassiliadis (RV; Becton Dickinson, Sparks, MD, USA) media. After overnight incubation at 42 °C in both of these enrichment broths, one loopful from each enrichment broth was spread on two different differential media: Brilliant Green Sulfa with novobiocin (BGS; Becton Dickinson, Sparks, MD, USA) agar and xylose lysine tergitol-4 (XLT-4; Becton Dickinson, Sparks, MD, USA) agar. These plates were incubated overnight at 35 °C, and on each plate, 3 *Salmonella*–like colonies per subsample were picked and confirmed using triple sugar iron agar (TSI; Becton Dickinson, Sparks, MD, USA) and lysine iron agar fermentation (LIA; Becton Dickinson, Sparks, MD, USA) using an incubation period of 18–24 h at 35 °C. Final confirmation of suspect TSI/LIA isolates was performed using *Salmonella* polyvalent O antiserum agglutination (Becton Dickinson, Sparks, MD, USA), using the manufacturer’s specifications. Positive *Salmonellae* were serogrouped using individual *Salmonella* poly O antisera for O groups A through I, following the Kauffman-White scheme [58].

#### Campylobacter spp

Recovery of *Campylobacter* spp. from homogenized samples was performed as previously described [59]. Initially, 100 mL of homogenized suspension was removed, plated onto Campy–Cefex agar and incubated at 42 ± 1 °C for 36 to 48 h in a microaerobic atmosphere (5% O_2_, 10% CO_2_, 85% N_2_). Putative *Campylobacter spp.* colonies were enumerated, and up to five colonies per sample were subcultured on Brucella agar supplemented with 10% lysed horse blood (BAB plates) for isolation and incubated as previously described.

#### Listeria spp

As a pre-enrichment step, the stomached homogenates remained in the filtered stomacher bags and were incubated overnight at 35 °C. This pre-enrichment was followed by two subsequent enrichments in UVM Modified Listeria Enrichment Broth (UVM, Becton Dickinson, Sparks, MD, USA) and Fraser Broth (Becton Dickinson, Sparks, MD, USA), both requiring an overnight incubation period at 30 °C. One loopful of the Fraser enrichment was streaked for isolation on *Listeria*-selective agar (Becton Dickinson, Sparks, MD, USA). These plates were incubated overnight at 30 °C, and on each plate, 3 *Listeria*–like colonies per positive subsample were picked and confirmed as *Listeria* using the appropriate BAX PCR assay (DuPont, Wilmington, DE, USA).

### Fecal and soil physiochemical analysis

The moisture content of the fecal and soil samples was determined by drying overnight at 65 °C and calculating the difference between the wet and dried weights of the litter. Fecal and soil pH and electrical conductivity (EC) were determined using an Orion Versa Star Advanced Electrochemistry Meter (Thermo Fisher Scientific, Waltham, MA, USA) and using a 1:5 dilution in distilled water. Fecal and soil samples were submitted to the University of Georgia Soils Testing Laboratory for Total C, Total N, and elemental (Al, B, Ca, Cd, Cr, Cu, Fe, K, Mg, Mn, Mo, Na, Ni, P, Pb, S, Si, Zn) composition.

### DNA extraction and quantification

DNA extractions were performed on 0.33 g of feces and 0.33 g of soil. DNA was extracted from samples according to a semi-automated hybrid DNA extraction protocol previously described [60]. This method was a combination of a mechanical method using the FastDNA Spin Kit for Feces (MP Biomedicals, Solon, OH, USA) and an enzymatic method based on the QIAamp DNA Stool Mini Kit (QIAGEN, Valencia, CA, USA). DNA purification was performed using the DNA Stool—Human Stool—Pathogen Detection Protocol of the QIAcube Robotic Workstation. After purification, the DNA concentration in each sample was determined spectrophotometrically using the Take3 plate in conjunction with the Synergy H4 multimode plate reader (BioTek, Winooski, VT, USA).

### Illumina MiSeq library construction and analyses

Library construction and sequencing were performed by the Earth Microbiome Project Laboratory at the U.S. Department of Energy, Argonne National Laboratory (Argonne, IL, USA). In short, the hypervariable V4 domain of the bacterial 16 S rRNA gene was amplified using the F515 (5′-CACGGTCGKCGGCGCCATT-3′) and R806 (5′-GGACTACHVGGGTWTCT AAT-3′) primer set with each primer containing Illumina adapter regions (Illumina, Inc., San Diego, CA, USA) and the reverse primer containing the Golay barcodes to facilitate multiplexing [61]. Raw reads were obtained by using the Illumina MiSeq platform. A total of 3,297,242 raw sequence reads were generated and processed by the QIIME v1.9.1 (Quantitative Insights Into Microbial Ecology) pipeline [62]. Quality filtering and library splitting according to the Golay barcode sequences were performed on the R1 read (split_library_fastq.py script, default parameters). Sequences were chimera checked against the gold.fa database (http://drive5.com/uchime/gold.fa) and clustered into Operational Taxonomic Units (OTUs) according to their sequence similarity (97%) using the usearch option [63] with pick_otus.py script (-m usearch, all other parameters were default). A representative sequence for each OTU was selected with pick_rep_set.py script (using the most abundant method for picking, all other parameters were default) and used for taxonomic assignment using UCLUST and the Greengenes 13_8 database [64] with assign_taxonomy.py (default parameters). Sequences were aligned (align_seqs.py script, default parameters) using PyNAST [65] and filtered (filter_alignment.py, default parameters). A phylogenetic tree was subsequently produced with the make_phylogeny.py script (with default settings and FastTree program). A total of 1,823 OTUs were identified among all the 1,393 samples collected (Feces_Start = 200, Feces_Mid = 313, Feces_End = 185, Soil_Start = 199, Soil_Mid = 313, Soil_End = 183). During the data preprocessing stage, the dimensions of the OTUs were reduced to 877 with unique genera.

### Model evaluation and prediction

In this study, we evaluated the performance of three machine learning algorithms (random forest (RF), support vector machine (SVM) and logistic regression (LogReg)) on our microbiome dataset as input with foodborne zoonotic poultry pathogens (*Salmonella*, *Campylobacter* and *Listeria*), farm management practices and physicochemical properties as targets. We dropped only microbiome genera columns belonging to *Salmonella*, *Campylobacter* and *Listeria* and selected all other microbiome genera as input features for our model building. Multiple binary classifiers were built separately for model prediction and evaluation. We used 5-fold cross-validation for all the models to train, evaluate and predict the data sampled from the dataset. In 5-fold cross-validation, the dataset is divided into five equal subsets (train on four subsets and evaluate on the remaining subset) and then does so repeatedly five times, changing the subset used for evaluation. This is done to reduce the impact of data variability and get more accurate representation on how well the model will perform on unseen data. The average prediction accuracy is computed by scoring metric of individual estimator algorithms (RF, LogReg, and SVM) in scikit-learn (v0.24.2) using cross-val-score function. AUCROC as a measure of trade-off between the “true positive rate” (sensitivity) and the “false positive rate” (1 - specificity) in class prediction was computed using roc_auc_score function in scikit-learn. AUROC with a value closer to 1.0 indicates a higher level of performance for an overall classification model. Data analysis, statistical tests and visualization were performed in Jupyter Notebook environment (v 6.0.3). Our analyses were performed using Python (v3.8.3), scikit-learn (v0.24.2), pandas (v1.2.5) and SciPy (v1.7.0) and results were presented by seaborn (v 0.10.1) heatmaps. Target variables include 3 pathogens (*Salmonella*, *Campylobacter* and *Listeria*), 24 physicochemical properties (pH, EC, Moisture, TotalC, TotalN, CNRatio, Al, B, Ca, Cd, Cr, Cu, Fe, K, Mg, Mn, Mo, Na, Ni, P, Pb, S, Si, Zn) and 32 farm management practices (AvgNumBirds, AvgNumFlocks, YearsFarming, EggSource, BroodBedding, BroodFeed, BrGMOFree, BrSoyFree, BrMedicated, BroodCleanFrequency, AvgAgeToPasture, PastureHousing, FreqHousingMove, AlwaysNewPasture, PastureFeed, PaGMOFree, PaSoyFree, PaMedicated, LayersOnFarm, CattleOnFarm, SwineOnFarm, GoatsOnFarm, SheepOnFarm, WaterSource, FreqBirdHandling, AnyABXUse, LengthFeedRestrixProcess, Seasons, FlockAgeDays, Breed, FlockSize, AnimalSource) as previously described [14, 15].

### Electronic supplementary material

Below is the link to the electronic supplementary material.

Supplementary Material 1

Supplementary Material 2

Supplementary Material 3

Supplementary Material 4

Supplementary Material 5

Supplementary Material 6

Supplementary Material 7

Supplementary Material 8

## Data Availability

All data analyzed during this study are included in this published article (and its supplementary information files).
