# Supplementary material for: Predicting foodborne pathogens and probiotics taxa within poultry-related microbiomes using a machine learning approach
Source: Anim Microbiome. 2023 Nov 15;5:57. doi: 10.1186/s42523-023-00260-w (PMC10648331; doi:10.1186/s42523-023-00260-w)
Supplement: Supplementary file 1 — Supplementary Material 1 [file 42523_2023_260_MOESM1_ESM.docx]

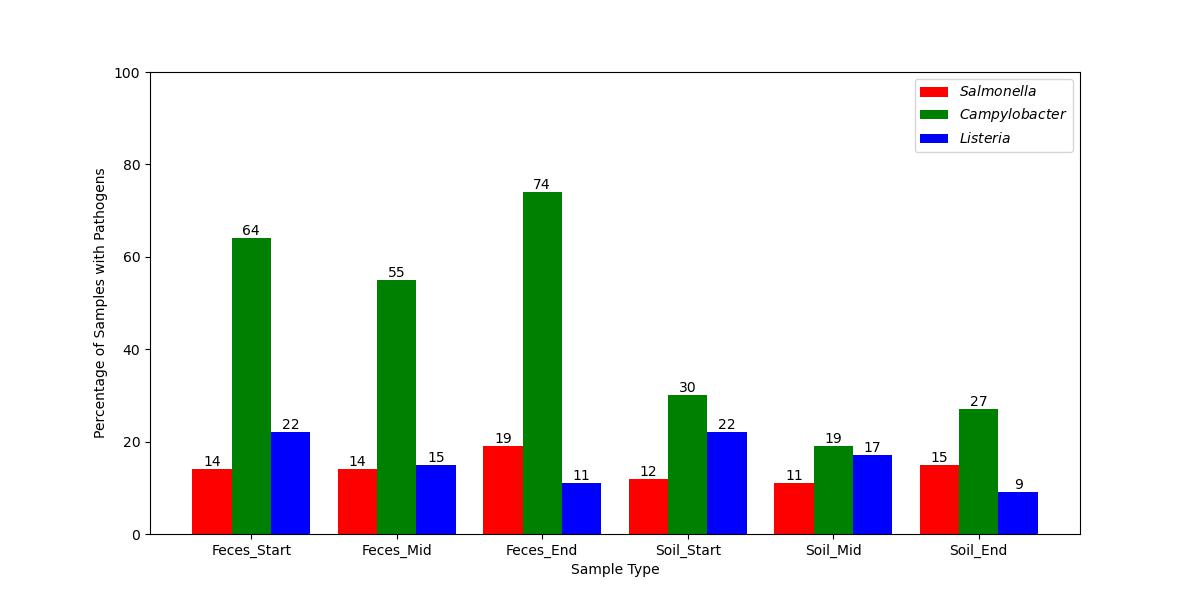


**Supplementary Figure 1:** Percentage pathogens species (*Salmonella* (red), *Campylobacter* (green), and *Listeria* (blue)) in total samples collected at varying stages (Start = 2-4 weeks, Mid= 5-7 weeks, End = 8-11 weeks) and different sample types (feces and soil) during pastured poultry operation. *Campylobacter* species is the most detected pathogen, followed by *Salmonella*, and *Listeria* is the least detected pathogen. *Campylobacter* detection was relatively higher at the beginning and end of the grow-out phase than at the middle stage of sample collection in both feces and soil. Prevalence of *Listeria* gradually declines as a function of time in feces and soil during the grow-out phase. *Salmonella* percentage detection remains fairly constant during the entire grow-out, processing and storage phases.

**Table 2:** 5-fold cross validation results with random forest (RF), support vector machine (SVM) and logistic regression (LogReg) for poor performing models (5-fold cross-validation < 0.7).

| **Number** | **Target Variable** | **RF** | **SVM** | **LogReg** |
| --- | --- | --- | --- | --- |
|  | **Farm Practice Variables** | | | |
| 1 | AvgNumBirds | 0.56 | 0.35 | 0.51 |
| 2 | AvgNumFlocks | 0.61 | 0.33 | 0.52 |
| 3 | YearsFarming | 0.54 | 0.22 | 0.45 |
| 4 | EggSource | 0.62 | 0.58 | 0.58 |
| 5 | BroodFeed | 0.52 | 0.33 | 0.47 |
| 6 | BroodCleanFrequency | 0.69 | 0.62 | 0.62 |
| 7 | PastureFeed | 0.51 | 0.34 | 0.46 |
| 8 | LengthFeedRestrixProcess | 0.26 | 0.58 | 0.56 |
| 9 | Seasons | 0.61 | 0.55 | 0.55 |
| 10 | FlockAgeDays | 0.36 | 0.1 | 0.26 |
| 11 | Breed | 0.69 | 0.65 | 0.63 |
| 12 | FlockSize | 0.51 | 0.35 | 0.45 |
